# Development of safety protocols for managing thirst in post-extubated and tracheostomized patients*

**DOI:** 10.1590/1518-8345.7376.4497

**Published:** 2025-05-02

**Authors:** Isabela Bossi Faleiros, Meiriane Pizani Scobare de Oliveira, Aline Franco da Rocha, Lígia Fahl Fonseca

**Affiliations:** 1 Universidade Estadual de Londrina, Londrina, PR, Brazil

**Keywords:** Thirst, Airway Extubation, Tracheostomy, Clinical Protocols, Practice Guideline, Intensive Care Units

## Abstract

to develop two distinct protocols, based on evidence, with safety criteria for post-extubated and tracheostomized patients in order to support the administration of thirst relief methods.

methodological study divided into two phases: definition of the scope and development of the protocols. Safety criteria were identified and submitted to the evaluation of reliability of the evidence using the Grading of Recommendations, Assessment, Development and Evaluation (GRADEpro) system. The criteria were incorporated into flowcharts with their respective operational manuals. The Experts formulated recommendations and the Content Validity Index was calculated.

two protocols and its respective manuals were developed to assess the safety of the administration of thirst relief methods: one for post-extubated patients and the other for tracheostomized patients. Safety criteria listed: level of consciousness, signs of respiratory failure, nausea and/or vomiting, swallowing ability, coughing/airway protection, voice changes and adequate functioning of the tracheostomy tube. The Experts’ recommendations were obtained with a Content Validity Index of 87%.

the clinical protocols, with six safety criteria each, based on evidence for the management of thirst in post-extubated and tracheostomized patients are innovative, allow safety assessment for thirst management and shows content validity.

## Highlights

**(1)** Thirst is intense in post-extubated and tracheostomized patients, but it is not treated. **(2)** There are no protocols with safety criteria for managing thirst in this population. **(3)** Evidence-based protocols with six safety criteria have been developed. **(4)** Innovative protocols assist nurses in decision-making. **(5)** It enables assessment and management of thirst through six safety criteria.

## Introduction

Several populations of patients with fluid restriction suffer from untreated thirst. Patients admitted to the Intensive Care Unit (ICU), in particular, have a high prevalence of intense and uncomfortable thirst, ranging from 69.8%^(1)^ to 76.1%^(2)^. The main factors that trigger thirst include: use of opioids and diuretics, inhaled medications, serum sodium and glucose levels, oral intake capacity, fasting, surgeries, osmolarity and Mechanical Ventilation (MV)^(3-4)^.

Thirst in critically ill patients is prevalent and challenging to manage; yet, paradoxically, it is often neglected^(5-7)^. The team relates thirst mainly to osmotic and volumetric factors (homeostatic thirst), directing its treatment only to intravenous rehydration. Non-homeostatic thirst, however, is triggered by dryness stimuli originating from the oropharynx, among others^(8-9)^, which is particularly relevant for its management in post-extubated and tracheostomized patients.

Thirst plays a key role in the perception of stress and suffering of ICU patients. The use of devices such as the orotracheal tube (OTT) and the need for MV are part of daily life, which causes the oral cavity remaining open open and exposed for prolonged of time. In view of this, patients report their experience of thirst as an overwhelming and constant sensation, having manifested itself as prevalent^(10)^ and intense^(5)^, the main and most frightening stressor^(11)^, distressing symptom^(12)^ and the second greatest discomfort in the ICU^(13)^. When the patient is conscious and on MV, this means that he or she is painfully aware of the discomfort caused by thirst^(14)^.

As intensive care becomes effective, the patient improves, culminating in extubation or replacement of the OTT with a long-term device, in this case, a tracheostomy (TQT)^(15)^. After extubation or while maintaining a TQT on MV, it is clinical practice to fast the patient from solids and liquids due to the risk of dysphagia.

This is a time when the patient, after prolonged intubation, complains persistently of thirst, making it urgent to manage this discomfort. There is no established consensus regarding the ideal time to perform tests to confirm the presence of dysphagia, with practices ranging from 6 to 24 hours of fasting. However, these procedures lack standardization and do not follow clear guidelines based on evidence^(16)^.

The most concerning complication when considering the early introduction of liquid and solid foods is post-extubation dysphagia (PED), which affects 41% of critically ill adults. It contributes to increased pneumonia rates, prolonging ICU stays and generating higher healthcare costs^(17)^.

It is necessary to develop protocols that counter dysphagia and make it possible to relieve thirst in critically ill patients. Some strategies using cold and mentholated substances^(1,18-25)^ have shown effectiveness in relieving thirst in critically ill patients. However, no protocols have been identified in literature that allow ICU nurses to assess the safety of using strategies to manage thirst, particularly for recently extubated and tracheostomized patients.

The development of a clinical protocol based on solid evidence was essential to guide care, enabling early management of thirst in these two populations. Evidence-Based Practice (EBP) represents a methodological approach that promotes the use of effective and successful interventions in clinical practice, by integrating the best scientific evidence from research, clinical experience and relevance to the patient, with the aim of supporting decision-making^(26)^.

Although there are effective strategies to alleviate thirst, it is essential, before using any relief method, to determine the safety criteria, especially in recently extubated patients or those with tracheostomy. Given the experiences in clinical settings and gaps in the literature, the need to develop a protocol based on scientific evidence that allows the safe and early use of methods to relieve thirst became evident. The objective of this study was to develop, based on evidence, two distinct protocols with safety criteria for patients with OTT and TQT in order to support the administration of thirst relief methods.

## Method

### Study design

This is a methodological study with a quantitative approach. The development of clinical protocols is based on robust and well-founded data, resulting in reliable and validated information^(27)^.

As a methodological reference, the manual “Methodological Guidelines: development of clinical guidelines” from the Ministry of Health was used, which consists of two phases: scope and development. The first consists of defining the theme, the working group and constructing the evidence search strategy. The second phase, in turn, includes the implementation of the search strategy, extraction, analysis of evidence, expert opinion for evidence analysis and development of recommendations using the Delphi Method and, finally, the development of the evidence-based guideline itself^(27)^.

### Participants

To develop the protocols, different groups were created to organize the process, define the scope, coordinate the work team, write the document, summarize the evidence, and develop recommendations. These groups included individuals with experience in the field and experts who determined the evidence. Thus, to conduct the following groups were formed to conduct the research: Coordinating Committee, Development Group, and Expert Group^(27)^.

The Coordinating Committee (CC) was composed of the principal investigator, her advisor and co-advisor, who were responsible for operationalizing the process. The Development Group (DG) was formed by the union of the CC and the secondary researcher, who were responsible for applying the search strategy and developing the guideline. The Expert Group (EG) was composed of intensive care nurses, physicians and physiotherapists, speech therapists, and nursing technicians, who were responsible for participating in the definition of recommendations, assisting in the interpretation of results, formulating suggestions, and reviewing the content of the guideline.

To select the EG, health professionals with more than three years of experience in intensive care were considered. By physical letter and electronic means, 17 professionals were invited, including seven nurses, two physiotherapists, one speech therapist, five doctors and two nursing technicians. Sixteen professionals participated in the research, with one nursing technician declining the invitation.

### Theme definition and search strategy

The theme defined for the guideline was safety for managing thirst in post-extubated and tracheostomized patients in the ICU. The topic was established based on the demand noted by the authors and requests to the Thirst Study and Research Group (GPS), based at the *Universidade Estadual de Londrina* (UEL). After the theme was proposed, the guideline was prepared in two stages, following the proposed methodological framework. The first was to list and analyze the evidence through a systematic review (SR) with meta-analysis entitled “Prevention of bronchoaspiration in the management of thirst in critically ill patients: systematic review and meta-analysis”. The SR followed the Preferred Reporting Items for Systematic Reviews and Meta-Analyses (PRISMA) methodology^(28)^ and received the registration number CRD42023429760 on the PROSPERO platform, where registration data indicate the methodological rigor of the search protocol. The review article, however, generated extensive results and therefore cannot be included in this construct. The search was carried out on June 14, 2023. The selected articles obtained a low risk of bias using the Rob 2^(29)^ and Robins I^(30)^ tools and high certainty of evidence using the GRADEpro software^(31)^. Under methodological rigor, it was possible to list six safety criteria for the elaboration of an evidence-based guideline for the management of thirst in post-extubated and tracheostomized patients, namely: level of consciousness, airway protection through the ability to swallow and cough, pulse oximetry, and absence of nausea/vomiting.

### Protocol creation

Even though they were not catalogued by the SR articles, the following were added to the criteria described: voice alteration and tracheostomy dysfunction. The first, because it is included in dysphagia screening and food reintroduction protocols; the second, due to the high risk of adversities due to possible anomalies in the TQT.

Once the criteria were selected, the second stage of the study was carried out by the preparation of two flowcharts and their respective operational manuals. The protocols were called SEDE-E (extubated) and SEDE-T (tracheostomized) to differentiate the flowchart in relation to the target population. It was decided to develop two protocols due to the specificity of each clinical situation: recently extubated patients do not have difficulty swallowing due to the permanent airway, and tracheostomized patients have a lower risk of bronchoaspiration due to the protection of the inflated cuff.

### Validation and recommendation of evidence

For content validation, separate instruments were used for the two flowcharts. In the first instrument, Flowchart Content Validation, five dimensions were assessed: general impressions about the flowchart, layout, flowchart content, applicability and relevance of the flowchart content to the operational manual. Each dimension was composed of items that explained what should be assessed in it, and the Experts made judgments about each of the items. This tool was adapted from the domains of the Appraisal of Guidelines for Research & Evaluation, AGREE II^(32)^. The responses comprised a five-point Likert scale: strongly disagree, disagree, neither agree nor disagree, agree, and strongly agree, followed by free considerations and suggestions from the Experts. The items assessed in the dimensions were used to calculate the Content Validity Index (CVI), with the scores agree and strongly agree being considered favorable. The second instrument, Operational Manual Content Validation, consisted of evaluating each item in the manual regarding its objectivity (allows for a specific response), clarity (clearly, simply and unequivocally explained) and relevance (evaluates the safety for managing thirst), followed by suggestions for reformulation. Each item in the tool was used to calculate the CVI, and a “yes” was considered favorable for each judgment.

The third instrument was the Judgment of the Intervention Recommendation. This tool consisted of several items whose objective was to help the expert make his/her recommendation. At the end of the evaluation, the recommendation could be judged according to the GRADEpro recommendations: strong recommendation against the intervention, conditional recommendation against the intervention, conditional recommendation for the intervention or comparison, conditional recommendation for the intervention, strong recommendation for the intervention^(31)^. The conditional recommendation for the intervention and the strong recommendation for the intervention were considered favorable.

The fourth and final step was the socio-occupational characterization of the Experts regarding: age, profession, length of service, level of education, type and complexity of the institution in which they work, and field of activity. The information was used to outline the profile of the participants. Once the elaboration of the flowcharts and operational manuals was completed, the respective files were sent electronically to the experts — after they accepted to participate —, with the appropriate instructions from the authors regarding reading and development of the evaluation. The author remained available to clarify doubts that did not involve an impact on methodological rigor. To reach consensus among them, the Delphi Method^(27)^ was used, in one round, in the period of July 2023. The experts’ feedback was tabulated in an Excel^®^ spreadsheet (Version 2308). The calculation of the CVI was determined considering the representativeness of each item and should reach an agreement rate of 80%^(33)^, a condition for the CC to accept the pertinent suggestions.

After the Experts’ suggestions, a speech therapist specializing in dysphagia with fifteen years of experience in the ICU was consulted. Her suggestions, added to those of the experts, contributed to the formulation of a process to directly and systematically assess swallowing ability. In addition, after the changes were made, an external technical consultation was held with the nursing coordinator and the medical head of the intensive care unit of the study institution, who validated all the changes summarized by the CC, as established in the Guidelines Manual^(27)^.

The methodology proposed by the manual includes consultation with Experts in order to obtain group consensus regarding the interpretation of the available evidence, although the manual advises that, in the hierarchy of evidence in health decision-making, the opinion of experts is considered a low level of evidence because it is more subject to bias. It also recommends the GRADE system because it offers a comprehensive and transparent methodology for structured decision-making, which is the methodology preferred by the Ministry of Health^(27)^.

Thus, the objective of the consultation with the Experts was to recommend the evidence already raised, with a strong indication of approval. Since the evidence passed the GRADEpro^(31)^ scrutiny and did not demonstrate bias using the Robins I^(30)^ and Rob 2^(29)^ tools, all suggestions were accepted, and the protocol was recommended with an 87% CVI index. The CC then considered that a new Delphi round of evaluation with the EG was not necessary.

## Ethical aspects

The study complied with the ethical aspects of research with human beings, as determined by Resolution CNS 466/12. The research project was submitted to the Research Ethics Committee (UEL) and obtained ethical approval under the number CAAE: 65456622.2.0000.5231.

## Results

**Figure 1 -:**
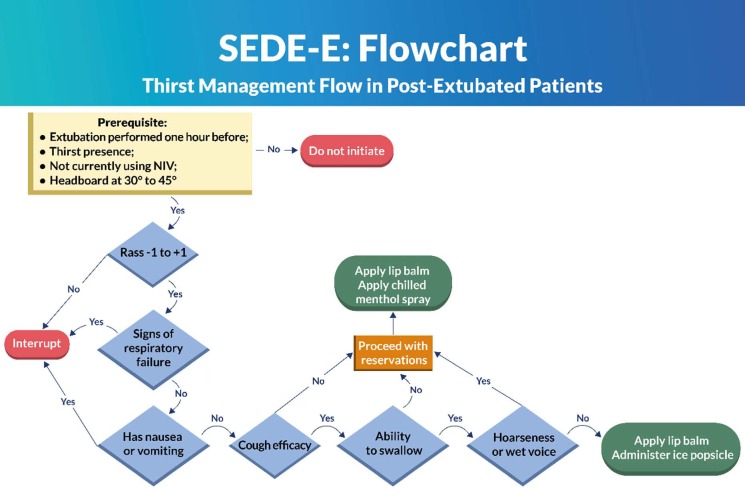
SEDE-E: Thirst management flow in post-extubated patients. Londrina, PR, Brazil, 2023

**Figure 2 -:**
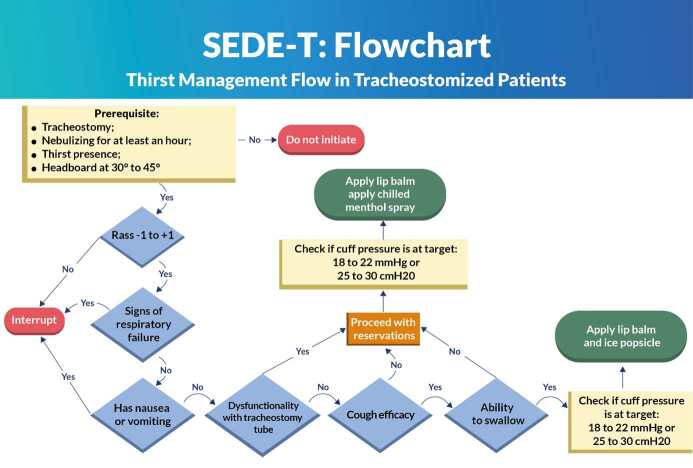
SEDE-T: Thirst management flow in tracheostomized patients. Londrina, PR, Brazil, 2023

Two evidence-based flowcharts were developed to manage thirst, taking into account the different characteristics of each patient group. The first flowchart was aimed at post-extubated patients, SEDE-E (Figure 1); the other, at tracheostomized patients, SEDE-T (Figure 2). Post-extubated patients no longer have difficulty swallowing related to the OTT and its cuff; tracheostomized patients, on the other hand, continue to have the tracheal cannula and its inflated cuff.

The flowcharts were prepared with their respective operational manuals. The manuals assist in the application of the protocol and explain each safety criterion, guiding decision-making.

The professions of the sixteen expert participants were: seven nurses, two physiotherapists, one speech therapist, five physicians and one nursing technician. The sociodemographic characteristics of the experts are described in Table 1.

**Table 1 -:** Distribution of sociodemographic and professional characteristics of study participants (n = 16). Londrina, PR, Brazil, 2023

**Variables**	**n**	**%**
**Professional training**		
Nursing	07	43.7
Medicine	05	31.2
Physiotherapy	02	12.5
Speech Therapy	01	06.3
Nursing Technician	01	06.3
**Educational background**		
Technical training	01	06.3
Post-graduate course	04	25.0
Master’s	05	31.2
Doctorate	06	37.5
**Size of the work institution**		
Special (over 500 beds)	03	18.7
Large (151-500 beds)	12	75.0
Medium (51–150 beds)	01	06.3
**Age**		
Up to 31 years	01	06.3
Between 31 and 40 years	08	50.0
Between 41 and 50 years	03	18.7
Over 50 years	04	25.0
**Time of professional activity**		
Between 5 and 10 years	03	18.7
Between 11 and 15 years	03	18.7
Between 16 and 20 years	01	06.3
Between 21 and 25 years	02	12.5
Between 26 and 30 years	01	06.3
Over 31 years	04	25.0
No answer	02	12.5

The Content Validation of the Flowchart for SEDE-E showed that the item on the order of the criteria reached an agreement CVI of 0.75; in view of this, the order of the criteria was changed to a more logical order. The dimension on the content of the operational manual, both in SEDE-E (0.68) and in SEDE-T (0.62), did not reach 80% agreement, therefore changes were suggested to improve the swallowing assessment (Table 2).

**Table 2 -:** Content validity index of the instrument validating the content of the flowchart and operational manual. Londrina, PR, Brazil, 2023

**Dimension**	CVI* SEDE-E	CVI* SEDE-T
CVI* Flowchart Content Validation		
**General Impressions**		
Its use is easy	1.00	1.00
It is self-explanatory	1.00	1.00
It is educational	1.00	1.00
**Flowchart Layout**		
The visual composition is attractive and well organized	0.87	0.87
The order of the safety criteria is appropriate	0.75	0.87
The flowchart is easy to read	0.93	1.00
The colors used in the flowchart design are relevant	0.93	0.93
**Flowchart content**		
The safety criteria indicated in the diamonds of the flowchart are clear	0.81	0.81
The safety criteria are presented in an organized manner in a correct sequence	0.87	0.81
**Flowchart applicability**		
The flowchart recommendations have practical applicability	0.87	0.81
**Operational Manual Contents**		
The content is consistent with the flowchart	0.93	0.87
The information is adequate	0.75	0.81
The information is sufficient for the use of the flowchart	0.68	0.62
CVI* Operational Manual Content Validation		
**Prerequisites**		
Objective	1.00	0.87
Clear	0.93	0.62
Relevant	0.87	0.81
**Preliminary examination**		
Objective	1.00	1.00
Clear	0.87	0.93
Relevant	1.00	1.00
**Level of consciousness**		
Objective	1.00	1.00
Clear	1.00	1.00
Relevant	0.81	0.81
**Pulse oximetry**		
Objective	1.00	1.00
Clear	0.93	0.93
Relevant	0.81	0.81
**Nausea and/or vomiting**		
Objective	1.00	1.00
Clear	0.93	0.93
Relevant	1.00	1.00
**Effective swallowing**		
Objective	0.87	0.87
Clear	0.75	0.81
Relevant	0.87	0.87
**Voice change**		
Objective	0.93	-
Clear	0.87	-
Relevant	1.00	-
**Dysfunction with tracheostomy tube**		
Objective	-	0.93
Clear	-	0.87
Relevant	-	0.93
**Effective coughing and/or throat clearing**		
Objective	0.93	0.93
Clear	0.93	0.87
Relevant	1.00	0.93
**Thirst management strategy**		
Objective	1.00	0.93
Clear	0.93	0.87
Relevant	0.93	0.93

*IVC = Content Validity Index

The Operational Manual Content Validation instrument received a satisfactory evaluation, with only one item below 80% in the CVI, in SEDE-E, on the clarity of how to assess swallowing; improvements in the way of assessing swallowing were suggested. Regarding the SEDE-T evaluation in this instrument, the item clarity in the prerequisites obtained a value of 0.62, which was due to a layout error in the explanation in this manual; the suggestion was accepted, and the flowchart was corrected (Table 2).

The result of the last instrument – Judgment of the Intervention Recommendation – reached a CVI of 87% in favor of the intervention with the SEDE-E and SEDE-T protocols. In view of this, the EG recommended the guideline developed, with no need for further adjustments.

**Figure 3 -:**
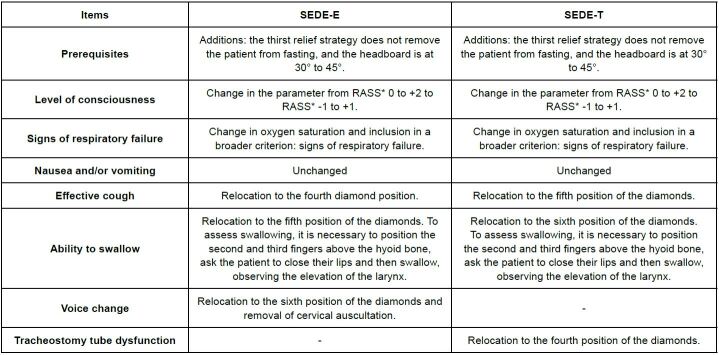
Changes made to the SEDE-E and SEDE-T protocols after suggestions from experts. Londrina-PR, Brazil, 2023 {#tbl:3}

After calculating the CVI of the assessment instruments, the Experts’ suggestions were evaluated. Therefore, the suggestions were gathered and accepted when pertinent (Figure 3). The external consultation with the nursing and medical coordinators of all the ICUs at the host institution resulted in consensus and agreement regarding the points, with the CVI reaching an index greater than 80% in all items.

*RASS = Richmond Agitation-Sedation Scale

## Discussion

The novelty of this study consists in the development, based on scientific evidence, of two clinical safety protocols for thirst management: one for post-extubated patients and the other for tracheostomized patients, in an innovative approach. They allow for the evaluation, through safety criteria, of the possibility of nurses applying, within less than 24 hours of extubation and even in tracheostomized patients, a strategy for relieving thirst safely, opening space for early treatment of thirst in patients who suffer intense distress due to the water restriction imposed on them during this therapeutic phase^(27)^.

Since it is not the most widely used method for developing clinical protocols, it was difficult to find a content validation instrument for both the flowcharts and the operational manuals. Therefore, the evaluation was carried out using a tool based on the domains of the Appraisal of Guidelines for Research & Evaluation, AGREE II, which assesses the methodological rigor and transparency of guidelines. Furthermore, the Experts were assisted by the GRADEpro tool to formulate the recommendation judgment^(31-32)^.

The safety criteria listed are in line with other protocols that aim to screen for dysphagia, such as the Gugging Swallowing Screen Intensive Care Unit (GuSS-ICU)^(34)^. Although it is not the focus of this research, avoiding bronchoaspiration is a common goal. The flowchart prepared begins with the prerequisites, with the criterion of an elevated headboard of 30° to 45°. After the Delphi round, two experts suggested that this be an exclusion criterion, because there are patients who are unable to raise the headboard, and therefore are more at risk of pulmonary aspiration.

The level of consciousness is, in most cases, the first criterion to be assessed. Other studies that aimed to relieve thirst^(1,20-21,35)^ or assess dysphagia^(34)^ in critically ill patients considered the assessment of this parameter through simple cognitive questions^(1,20,35)^, Richmond Agitation-Sedation Scale (RASS)^(21,34)^ or Glasgow Coma Scale (GCS)^(21)^. Being awake and alert, communicating, but not necessarily oriented, does not prevent the patient from receiving strategies to relieve thirst. In this item, the use of the GCS was suggested, but the CG, with the support of the institution’s medical and nursing managers, considered expanding the RASS parameter, which would cover the level of consciousness criterion with sufficient safety.

As suggested by the experts, pulse oximetry was included in a broader criterion – respiratory failure – to provide greater safety in the assessment. The following were also included in this criterion: dyspnea, use of accessory muscles (intercostal retraction, suprasternal and abdominal retraction), oxygen saturation <92% and presence of laryngeal stridor. Respiratory rate, oximetry and presence of stridor are observed as relevant criteria in other protocols that evaluate dysphagia, but not with this specific nomenclature^(34)^.

The absence of nausea and vomiting was considered an essential criterion aiming at the prevention of adverse events after the intervention to relieve thirst. Broncho aspiration is the most feared complication referenced by studies that evaluate the reintroduction of liquids and solids^(1,20-21,34)^, also being part of a safety protocol aimed at managing thirst in surgical patients^(36)^. This criterion was fully agreed upon by the EG.

Coughing is essentially an airway protection mechanism^(35)^. This resource ensures that if there are secretions or foreign bodies in the upper airways, they will be expelled. When it comes to managing thirst, effective coughing will ensure that the strategy used to relieve thirst does not obstruct the airways or lead to broncho aspiration. Clearing the throat intentionally and vigorously using a characteristic movement is considered sufficient to protect the airway. This criterion has been identified in studies aimed at managing thirst and reintroducing food^(1,21,34-35)^. All experts agreed on this item.

Similar to coughing, swallowing is an extremely important airway protection mechanism, especially for the purpose of the present study. A meta-analysis of the SR showed that 41% of post-extubated patients had dysphagia, causing pulmonary aspiration, without differentiating between short (<48 hours) or prolonged (>48 hours) intubation^(17)^. Screening for dysphagia for the purpose of reintroducing food is considered essential as a protective measure^(34)^. Managing thirst differs from introducing food, that is, it does not take the patient off the fast, since its objective is to safely alleviate the discomfort of thirst through cold and mentholated substances with low volume.

The experts presented important suggestions regarding the evaluation of the swallowing criterion. Therefore, after a meeting with the speech therapist, it was decided that swallowing would be evaluated by elevating the hyoid bone together with asking the patient to close their lips. During the swallowing process, the hyoid bone, which is above the laryngeal cartilage, presents an upward and anterior elevation movement^(36)^. In this way, it is possible to assess, with the second and third fingers placed above this bone, laryngeal elevation and, consequently, swallowing^(37)^.

The last criterion, in the case of SEDE-E, is voice change. Although it was not listed in the articles of the SR carried out, protocols that aim to assess the return of oral feeding consider voice change relevant^(34)^.

The SEDE-T did not include voice changes due to the presence of a tracheostomy tube, but the criterion of tube dysfunction was introduced. When there is tube dysfunction in patients using TQT, they are more prone to adverse events or a worse prognosis. Cuff leaks, cuff pressure above the recommended level, bleeding, poor positioning, and noncompliance with the ostomy are some examples^(37)^. All experts agreed on this item.

The strategies suggested for managing thirst were a 20-milliliter ice popsicle with or without menthol and a cold menthol spray, both together with menthol moisturizer. The effectiveness of the ice popsicle with or without menthol has been demonstrated in several Randomized Clinical Trials (RCTs)^(23,38-39)^, and the popsicle has been used extensively by patients in the Post Anesthesia Care Unit^(39)^. Just one mentholated ice popsicle reduces the intensity of thirst by 80.6% and discomfort by 75.9%^(39)^. It is worth noting that the use of ice helps in the recovery of dysphagia by reactivating the swallowing muscles^(40)^.

The cold temperature and menthol activate oropharyngeal receptors, which send signals directly to the terminal lamina, causing a decrease in the desire to drink water in approximately two minutes^(9,41-43)^. In other words, without large volumes of liquid, thirst is quenched more quickly. Another advantage is that the popsicle melts slowly, which increases the safety of thirst relief compared to drinking larger volumes of water.

The cold and mentholated spray uses the same principles of activating oral thermoreceptors and can be used in patients who are unable to cough or swallow, have voice changes or have dysfunctional TQT cannula. A spray-based thirst relief strategy is safe because it is easy to use and evenly distributes small droplets of water (0.1 milliliter) on the oral mucosal surface. This moistens the oral mucosa, controlling the amount of liquid introduced and regulating the amount of liquid applied, which in turn reduces possible adverse reactions, thus providing a high degree of safety^(1)^.

After modifications and approval by two doctors specializing in ICU, the SEDE-E and SEDE-T flowcharts were completed. Both protocols are a way for post-extubated and tracheostomized patients to relieve the distressing and intense symptom of thirst through simple management.

A limitation of the study is that only one round of the Delphi Technique was performed, although this weakness was overcome by consensus with another group of evaluators, from medicine and nursing, with extensive experience and expertise in intensive care. The group of experts predominantly from two hospitals in the South and Southeast of Brazil is a limitation, as other regions of the country may have different perspectives. Future evaluations of the protocols in other settings will help test them in different populations and contexts. Another limitation was the scarcity of studies addressing thirst management in tracheostomized patients, particularly focusing on the assessment of safety criteria, reducing the possibility of comparisons. It is suggested that the application of the protocols be evaluated in different settings, considering intervening and outcome variables, as well as the benefits perceived by the patient and the health team.

The present study contributes to the practice of intensive care nurses, assisting in decision-making and allowing them to safely alleviate thirst in critically ill patients, based on scientific evidence. In addition, it highlights the need for studies on this topic and for the evaluation of protocols in clinical practice. Finally, it not only encourages professionals to focus intentionally on the thirst of recently extubated and tracheostomized patients, but also provides two instruments that can make it possible to safely alleviate thirst and suffering in critically ill patients.

## Conclusion

The development of two evidence-based clinical protocols aimed at the safety of thirst management in critically ill patients who have been extubated and tracheostomized represents an innovation and a step forward in humanized care. These protocols propose six safety criteria each: level of consciousness, signs of respiratory failure, nausea and/or vomiting, effective cough, ability to swallow and voice alteration, and, in the case of tracheostomy, cannula dysfunction. Presented in easy-to-understand flowcharts and accompanied by an operational manual, the newly developed protocols, in addition to having content validity, open doors for the assessment of safety for relieving thirst in critically ill patients, contributing to the reduction of distress related to this symptom.
