# Supplementary material for: Development of safety protocols for managing thirst in post-extubated and tracheostomized patients
Source: Rev Lat Am Enfermagem. 2025 May 2;33:e4497. doi: 10.1590/1518-8345.7376.4497 (PMC12052312; doi:10.1590/1518-8345.7376.4497)
Supplement: Translation

# Translation: Elaboração de protocolos de segurança para manejo da sede em pacientes pós-extubados e traqueostomizados*

**DOI:** 10.1590/1518-8345.7376.4498

**Authors:** Isabela Bossi Faleiros, Meiriane Pizani Scobare de Oliveira, Aline Franco da Rocha, Lígia Fahl Fonseca

**Affiliations:** 1 Universidade Estadual de Londrina, Londrina, PR, Brasil

**Keywords:** Sede, Extubação, Traqueostomia, Protocolos Clínicos, Guia de Prática Clínica, Unidade de Terapia Intensiva

## Abstract

elaborar, com base em evidências, dois protocolos distintos com critérios de segurança para pacientes pós-extubados e traqueostomizados a fim de sustentar a administração de métodos de alívio da sede.

estudo metodológico dividido em duas fases: definição do escopo e elaboração dos protocolos. Identificaram-se critérios de segurança que foram submetidos à avaliação de confiabilidade das evidências pelo sistema *Grading of Recommendations, Assessment, Development and Evaluation* (GRADEpro). Os critérios foram incorporados em fluxogramas com seus respectivos manuais operacionais. Os painelistas formularam recomendação e calculou-se o Índice de Validade de Conteúdo.

desenvolveram-se dois protocolos e respectivos manuais para avaliação de segurança na administração de métodos de alívio da sede: um para pacientes pós-extubados e outro para traqueostomizados. Critérios de segurança elencados: nível de consciência, sinais de insuficiência respiratória, náusea e/ou vômito, capacidade de deglutição, tosse/proteção das vias aéreas, alteração de voz e funcionamento adequado da cânula de traqueostomia. Obteve-se recomendação dos painelistas com Índice de Validade de Conteúdo de 87%.

os protocolos clínicos, com seis critérios de segurança cada, com base em evidências para o manejo da sede em pacientes pós-extubados e traqueostomizados são inovadores, permitem avaliação da segurança para o manejo da sede e têm validade de conteúdo.

## Destaques

**(1)** A sede é intensa em pacientes pós-extubados e traqueostomizados, porém não tratada. **(2)** Não há protocolos com critérios de segurança para manejar a sede nesta população. **(3)** Foram elaborados protocolos baseados em evidências com seis critérios de segurança.**(4)** Os protocolos inovadores auxiliam na tomada de decisão do enfermeiro. **(5)** Possibilitam, por meio de seis critérios de segurança, avaliação e manejo da sede.

## Introdução

Diversas populações de pacientes em restrição hídrica sofrem com sede não tratada. Os pacientes internados em Unidade de Terapia Intensiva (UTI), em particular, apresentam, com intensidade e desconforto, a sede com elevada prevalência, variando de 69,8%^(1)^ a 76,1%^(2)^. Os principais fatores para seu desencadeamento incluem: uso de opioides e diuréticos, medicamentos inalatórios, níveis séricos de sódio e glicose, capacidade de ingestão oral, jejum, cirurgias, osmolaridade e Ventilação Mecânica (VM)^(3-4)^.

A sede no paciente crítico, além de prevalente, é de manejo desafiador; mesmo assim, paradoxalmente, com frequência é negligenciada^(5-7)^. A equipe relaciona a sede sobretudo a fatores osmóticos e volumétricos (sede homeostática), direcionando-se seu tratamento somente à reidratação endovenosa. A sede não homeostática, no entanto, é deflagrada por estímulos de ressecamento oriundos da orofaringe, entre outros^(8-9)^, o que é particularmente relevante para seu manejo em pacientes pós-extubados e traqueostomizados.

A sede ocupa um papel principal em relação à percepção de estresse e sofrimento de pacientes em UTI. O uso de dispositivos como o tubo orotraqueal (TOT) e a necessidade de VM fazem parte do cotidiano, o que faz com que a cavidade oral permaneça aberta e exposta por períodos prolongados. Em vista disso, os pacientes se referem à experiência de sede como uma sensação avassaladora e constante, manifestando-se como prevalente^(10)^ e intensa^(5)^, assim como o principal e mais assustador estressor^(11)^, sendo referido como um sintoma angustiante^(12)^ e o segundo maior desconforto na UTI^(13)^. Quando o paciente está consciente e sob VM, isso significa que se encontra dolorosamente ciente do desconforto causado pela sede^(14)^.

Conforme o cuidado intensivo se torna resolutivo, há uma melhora do paciente, o que culminará em sua extubação ou substituição do TOT por um dispositivo de longa permanência, no caso, a traqueostomia (TQT)^(15)^. Após a extubação ou durante a manutenção de uma TQT em VM, é prática clínica deixar o paciente em jejum de sólidos e líquidos devido ao risco de disfagia.

Esse é um momento em que, depois de estar entubado por longos períodos, o paciente queixa-se insistentemente de sede, tornando premente a necessidade de manejar esse desconforto. Não há consenso estabelecido quanto ao momento ideal para a realização de testes que confirmem a presença de disfagia, com práticas que variam de 6 até 24 horas de jejum. Entretanto esses procedimentos carecem de padronização e não seguem diretrizes claras fundamentadas em evidências^(16)^.

A complicação mais preocupante ao se considerar a introdução precoce de alimentos líquidos e sólidos é a disfagia pós-extubação (DPE), que afeta 41% dos adultos gravemente enfermos. Ela contribui para o aumento das taxas de pneumonia, prolongando a permanência na UTI e gerando maiores custos com cuidados de saúde^(17)^.

É necessário desenvolver protocolos que se contraponham à disfagia e tornem possível o alívio da sede em pacientes críticos. Algumas estratégias utilizando substâncias frias e mentoladas^(1,18-25)^ têm demonstrado efetividade no alívio da sede em pacientes críticos. Todavia não se identificou na literatura a existência de protocolos que possibilitem aos enfermeiros nas UTIs avaliar a segurança na utilização de estratégias para o manejo da sede, particularmente para os pacientes recém-extubados e traqueostomizados.

A elaboração de um protocolo clínico embasado em evidências sólidas foi essencial para orientar o cuidado, possibilitando o manejo precoce da sede nessas duas populações. A Prática Baseada em Evidências (PBE) representa uma abordagem metodológica que promove a utilização de intervenções eficazes e bem-sucedidas na prática clínica, ao integrar as melhores evidências científicas provenientes de pesquisas, experiência clínica e de relevância para o paciente, com o objetivo de embasar a tomada de decisão^(26)^.

Ainda que haja estratégias efetivas para amenizar a sede, é fundamental, antes da utilização de algum método de alívio, determinar os critérios de segurança, especialmente em pacientes recém-extubados ou com traqueostomia. Diante das experiências em ambientes clínicos e de lacunas na literatura, ficou notória a necessidade de se elaborar um protocolo pautado em evidências científicas que permita o uso seguro e precoce de métodos para aliviar a sede. O objetivo deste estudo foi elaborar, com base em evidências, dois protocolos distintos com critérios de segurança para pacientes com TOT e TQT a fim de sustentar a administração de métodos de alívio da sede.

## Método

### Delineamento do estudo

Trata-se de um estudo metodológico de abordagem quantitativa. A elaboração de protocolos clínicos se baseia em dados robustos e bem fundamentados, resultando em informações confiáveis e validadas^(27)^.

Como referencial metodológico, utilizou-se o manual “Diretrizes Metodológicas: elaboração de diretrizes clínicas”, do Ministério da Saúde, que é composto por duas fases: escopo e elaboração. A primeira consiste na definição do tema e do grupo de trabalho e na construção da estratégia de busca da evidência. A segunda fase, por sua vez, compreende realização da estratégia de busca, extração, análise das evidências, opinião de especialistas para análise de evidências e elaboração das recomendações por meio do Método Delphi e, por fim, a elaboração em si da diretriz baseada em evidências^(27)^.

### Participantes

Para desenvolver os protocolos, delimitaram-se diferentes grupos encarregados de organizar o processo, definir o escopo, coordenar a equipe de trabalho, redigir o documento, resumir as evidências e elaborar recomendações. Esses grupos incluíram indivíduos com experiência na área e especialistas que determinaram as evidências. Dessa forma, para conduzir a pesquisa, foram constituídos os seguintes grupos: Comitê Gestor, Grupo Elaborador e Grupo de Painelistas^(27)^.

O Comitê Gestor (CG) foi composto pelo pesquisador principal, seu orientador e coorientador, responsáveis pela operacionalização do processo. O Grupo Elaborador (GE) formou-se da união do CG com o pesquisador secundário, responsável por aplicar a estratégia de busca e elaborar a diretriz. O Grupo de Painelistas (GP) foi constituído por enfermeiros, médicos e fisioterapeutas intensivistas, fonoaudiólogo e técnicos de enfermagem, responsáveis por participar da definição de recomendações, auxiliar na interpretação de resultados, formular sugestões e revisar o conteúdo da diretriz.

Para a seleção do GP, considerou-se profissionais de saúde com experiência de mais de três anos em cuidados intensivos. Por carta física e meio eletrônico, 17 profissionais foram convidados, sendo sete enfermeiros, dois fisioterapeutas, um fonoaudiólogo, cinco médicos e dois técnicos de enfermagem. Participaram da pesquisa 16 profissionais, sendo que um técnico de enfermagem declinou do convite.

### Definição do tema e estratégia de busca

A temática definida para a diretriz foi segurança para o manejo da sede em pacientes pós-extubados e traqueostomizados em UTI. Estabeleceu-se o tópico a partir da demanda notada pelos autores e por solicitações ao Grupo de Estudo e Pesquisa da Sede (GPS), sediado na Universidade Estadual de Londrina (UEL). Depois de proposto o tema, a diretriz foi elaborada em duas etapas, seguindo o referencial metodológico proposto. A primeira foi elencar e analisar as evidências por meio de uma revisão sistemática (RS) com metanálise intitulada “Prevenção de broncoaspiração no manejo da sede em pacientes críticos: revisão sistemática e metanálise”.

A RS seguiu a metodologia *Preferred Reporting Items for Systematic Reviews and Meta-Analyses* (PRISMA)^(28)^ e recebeu o número de registro CRD42023429760 na plataforma PROSPERO, onde dados de cadastro indicam rigor metodológico do protocolo de busca. O artigo de revisão, todavia, gerou resultados extensos, não podendo, portanto, compor este construto. A busca foi realizada no dia 14 de junho de 2023. Os artigos selecionados obtiveram baixo risco de viés pelas ferramentas Rob 2^(29)^ e Robins I^(30)^ e alta certeza de evidência por meio do *software* GRADEpro^(31)^. Sob rigor metodológico, foi possível elencar seis critérios de segurança para a elaboração de uma diretriz baseada em evidências para o manejo da sede em pacientes pós-extubados e traqueostomizados, sendo eles: nível de consciência, proteção de vias aéreas por meio da capacidade de deglutir e tossir, oximetria de pulso e ausência de náusea/vômito.

### Elaboração do protocolo

Mesmo sem terem sido catalogados pelos artigos da RS, foram acrescentados aos critérios descritos: alteração de voz e disfuncionalidade de traqueostomia. O primeiro, por ser contemplado em protocolos de triagem de disfagia e reintrodução alimentar; o segundo, devido ao elevado risco de adversidades por possíveis anomalias na TQT.

Selecionados os critérios, seguiu-se para a segunda etapa do estudo, com a elaboração de dois fluxogramas e seus respectivos manuais operacionais. Os protocolos foram denominados SEDE-E (extubados) e SEDE-T (traqueostomizados) para a diferenciação do fluxograma em relação à população-alvo. Optou-se por elaborar dois protocolos em razão da especificidade de cada situação clínica: pacientes recém-extubados não têm dificuldade de deglutição por permanência de via aérea definitiva, e pacientes traqueostomizados apresentam menor risco de broncoaspiração devido à proteção do *cuff* insuflado.

### Validação e recomendação da evidência

Para validação de conteúdo, utilizaram-se instrumentos separados para os dois fluxogramas. No primeiro instrumento, Validação de Conteúdo do Fluxograma, foram avaliadas cinco dimensões: impressões gerais sobre o fluxograma, *layout*, conteúdo do fluxograma, aplicabilidade e pertinência do conteúdo do fluxograma com o manual operacional. Cada dimensão era composta por itens que explicitavam o que se deveria avaliar nela, e os painelistas fizeram julgamento a respeito de cada um dos itens. Essa ferramenta foi adaptada dos domínios do *Appraisal of Guidelines for Research & Evaluation,* AGREE II^(32)^. As respostas compreendiam uma escala Likert de cinco pontos: discordo plenamente, discordo, não concordo nem discordo, concordo, e concordo plenamente, seguidas de livres considerações e sugestões dos painelistas. Os itens avaliados nas dimensões foram utilizados para cálculo de Índice de Validade de Conteúdo (IVC), tendo-se considerado favoráveis os escores concordo e concordo plenamente.

O segundo instrumento, Validação de Conteúdo do Manual Operacional, consistiu em julgar cada item do manual quanto à sua objetividade (permite resposta pontual), clareza (explicitado de forma clara, simples e inequívoca) e pertinência (avalia a segurança para o manejo da sede), seguido de sugestões de reformulação. Cada item da ferramenta foi utilizado para cálculo de IVC, e considerou-se favorável “sim” para cada julgamento.

O terceiro instrumento foi o Julgamento da Recomendação da Intervenção. Esta ferramenta foi composta por vários itens cujo objetivo era auxiliar o painelista a fazer sua recomendação. Ao final da avaliação, a recomendação poderia ser julgada conforme preconiza o GRADEpro: forte recomendação contra a intervenção, recomendação condicional contra a intervenção, recomendação condicional para a intervenção ou a comparação, recomendação condicional para a intervenção, forte recomendação para a intervenção^(31)^. Foram consideradas favoráveis a recomendação condicional para a intervenção e a forte recomendação para a intervenção.

O quarto e último foi a caracterização sócio-ocupacional dos painelistas quanto a: idade, profissão, tempo de atuação, grau de escolaridade, tipo e complexidade de instituição em que trabalha e campo de atuação. As informações serviram para traçar o perfil dos participantes. Concluída a elaboração dos fluxogramas e manuais operacionais, os respectivos arquivos foram enviados por meio eletrônico aos painelistas — após aceite de participação —, com as devidas orientações dos autores quanto à leitura e ao desenvolvimento da avaliação. O autor permaneceu disponível para esclarecimento de dúvidas que não envolvessem impacto no rigor metodológico. Para alcance de consenso entre eles, utilizou-se o Método Delphi^(27)^, em uma rodada, no período de julho de 2023. As devolutivas dos painelistas foram tabuladas em planilha *Excel* ^®^ (Versão 2308). O cálculo do IVC foi determinado considerando-se a representatividade de cada item e deveria alcançar índice de concordância de 80%^(33)^, condição para que o CG acatasse as sugestões pertinentes.

Depois das sugestões dos painelistas, consultou-se uma fonoaudióloga especialista em disfagia, com experiência de quinze anos em UTI. Suas sugestões, acrescidas às dos painelistas, contribuíram para a formulação de um processo para avaliar, de forma direta e sistemática, a capacidade de deglutir. Além disso, após as alterações, fez-se consulta técnica externa com a coordenadora de enfermagem e a chefia médica da divisão de terapia intensiva da instituição do estudo, que validaram todas as alterações sintetizadas pelo CG, como estabelece o Manual de Diretrizes^(27)^.

A metodologia proposta pelo manual inclui consulta com painelistas com a finalidade de obter consenso do grupo em relação à interpretação das evidências disponíveis, ainda que o manual oriente que, na hierarquia das evidências na tomada de decisão em saúde, a opinião de especialistas é considerada nível baixo de evidências por estar mais sujeita a vieses. Recomenda, ainda, o sistema GRADE por oferecer uma metodologia abrangente e transparente para tomada de decisão estruturada, sendo essa a metodologia preferencialmente utilizada pelo Ministério da Saúde^(27)^.

Dessa forma, o objetivo da consulta aos painelistas foi a recomendação das evidências já levantadas, com forte indício de aprovação. Uma vez que as evidências passaram pelo crivo do GRADEpro^(31)^, não demonstraram vieses pelas ferramentas Robins I^(30)^ e Rob 2^(29)^, todas as sugestões foram acatadas e o protocolo foi recomendado com índice de 87% no IVC. O CG julgou, então, não ser necessária uma nova rodada Delphi de avaliação com o GP.

## Aspectos éticos

O estudo respeitou os aspectos éticos acerca de pesquisa com seres humanos, segundo determina a Resolução CNS 466/12. O projeto de pesquisa foi submetido à apreciação do Comitê de Ética em Pesquisa da Universidade Estadual de Londrina e obteve a aprovação ética sob o número CAAE: 65456622.2.0000.5231.

## Resultados

Desenvolveram-se dois fluxogramas baseados em evidências científicas visando ao manejo da sede, obedecendo às distintas particularidades dos grupos de pacientes. O primeiro fluxograma foi direcionado a pacientes pós-extubados, SEDE-E (Figura 1); o outro, a traqueostomizados, SEDE-T (Figura 2). Pacientes pós-extubados já não apresentam a dificuldade de deglutição relacionada ao TOT e seu *cuff*; os traqueostomizados, por sua vez, permanecem com a cânula traqueal e seu *cuff* insuflado.

Os fluxogramas foram elaborados com seus respectivos manuais operacionais. Os manuais auxiliam na aplicação do protocolo e explicitam cada critério de segurança, direcionando a tomada de decisão.

**Figura 1 -:** SEDE-E: Fluxo de manejo da sede no paciente pós-extubado. Londrina, PR, Brasil, 2023

**Figura 2 -:** SEDE-T: Fluxo de manejo da sede no paciente traqueostomizado. Londrina, PR, Brasil, 2023

Participaram 16 painelistas: sete enfermeiros, dois fisioterapeutas, um fonoaudiólogo, cinco médicos e um técnico de enfermagem. A caracterização sociodemográfica dos painelistas está descrita na Tabela 1.

**Tabela 1 -:** Distribuição das características sociodemográficas e profissionais dos participantes do estudo (n = 16). Londrina, PR, Brasil, 2023

**Variáveis**	**n**	**%**
**Formação profissional**		
Enfermagem	07	43,7
Medicina	05	31,2
Fisioterapia	02	12,5
Fonoaudiologia	01	06,3
Técnico de Enfermagem	01	06,3
**Formação educacional**		
Curso Técnico	01	06,3
Especialização	04	25,0
Mestrado	05	31,2
Doutorado	06	37,5
**Porte da instituição de trabalho**		
Especial (acima 500 leitos)	03	18,7
Grande Porte (151-500 leitos)	12	75,0
Médio Porte (51–150 leitos	01	06,3
**Idade**		
Até 31 anos	01	06,3
Entre 31 a 40 anos	08	50,0
Entre 41 a 50 anos	03	18,7
Mais que 50 anos	04	25,0
**Tempo de atuação profissional**		
Entre 5 e 10 anos	03	18,7
Entre 11 e 15 anos	03	18,7
Entre 16 e 20 anos	01	06,3
Entre 21 e 25 anos	02	12,5
Entre 26 e 30 anos	01	06,3
Mais que 31 anos	04	25,0
Não responderam	02	12,5

A Validação de Conteúdo do Fluxograma para o SEDE-E mostrou que o item sobre a ordem dos critérios atingiu IVC de 0,75 de concordância; em vista disso, alterou-se a ordem dos critérios para uma ordem mais lógica. A dimensão sobre o conteúdo do manual operacional, tanto no SEDE-E (0,68) quanto no SEDE-T (0,62), não atingiu 80% de concordância, portanto foram sugeridas alterações para melhorar a avaliação da deglutição (Tabela 2).

**Tabela 2 -:** Índice de validade de conteúdo do instrumento validação de conteúdo do fluxograma e manual operacional. Londrina, PR, Brasil, 2023

**Dimensões**	IVC* SEDE-E	IVC* SEDE-T
IVC* Validação de Conteúdo do Fluxograma		
**Impressões Gerais**		
Seu uso é fácil	1,00	1,00
É autoexplicativo	1,00	1,00
É didático	1,00	1,00
***Layout* do Fluxograma**		
A composição visual está atrativa e bem organizada	0,87	0,87
A ordem dos critérios de segurança está adequada	0,75	0,87
O fluxograma é de fácil leitura	0,93	1,00
As cores utilizadas no design do fluxograma são pertinentes	0,93	0,93
**Conteúdo do Fluxograma**		
Os critérios de segurança indicados nos losangos do fluxograma estão claros	0,81	0,81
Os critérios de segurança apresentam-se de forma organizada em uma sequência correta	0,87	0,81
**Aplicabilidade do Fluxograma**		
As recomendações do fluxograma têm aplicabilidade prática	0,87	0,81
**Conteúdo do Manual Operacional**		
O conteúdo é coerente com o fluxograma	0,93	0,87
As informações são adequadas	0,75	0,81
As informações são suficientes para o uso do fluxograma	0,68	0,62
IVC* Validação de Conteúdo do Manual Operacional		
**Pré-Requisitos**		
Objetivo	1,00	0,87
Claro	0,93	0,62
Pertinente	0,87	0,81
**Exame preliminar**		
Objetivo	1,00	1,00
Claro	0,87	0,93
Pertinente	1,00	1,00
**Nível de consciência**		
Objetivo	1,00	1,00
Claro	1,00	1,00
Pertinente	0,81	0,81
**Oximetria de pulso**		
Objetivo	1,00	1,00
Claro	0,93	0,93
Pertinente	0,81	0,81
**Náusea e/ou vômito**		
Objetivo	1,00	1,00
Claro	0,93	0,93
Pertinente	1,00	1,00
**Deglutição eficaz**		
Objetivo	0,87	0,87
Claro	0,75	0,81
Pertinente	0,87	0,87
**Alteração de voz**		
Objetivo	0,93	-
Claro	0,87	-
Pertinente	1,00	-
**Disfuncionalidade com cânula de traqueostomia**		
Objetivo	-	0,93
Claro	-	0,87
Pertinente	-	0,93
**Tosse e/ou limpar garganta eficaz**		
Objetivo	0,93	0,93
Claro	0,93	0,87
Pertinente	1,00	0,93
**Estratégia de manejo da sede**		
Objetivo	1,00	0,93
Claro	0,93	0,87
Pertinente	0,93	0,93

*IVC = Índice de Validade de Conteúdo

O instrumento Validação de Conteúdo do Manual Operacional obteve avaliação satisfatória, com apenas um quesito abaixo de 80% no IVC, no SEDE-E, sobre a clareza de como avaliar a deglutição; sugeriu-se melhora na forma de avaliar a deglutição. Quanto à avaliação do SEDE-T neste instrumento, o item clareza nos pré-requisitos obteve o valor 0,62, o que se deu pelo erro de diagramação da explicação neste manual; aceitou-se a sugestão e corrigiu-se o fluxograma (Tabela 2).

O resultado do último instrumento – Julgamento da Recomendação da Intervenção – alcançou IVC de 87% a favor da intervenção com os protocolos SEDE-E e SEDE-T. Em vista disso, o GP recomendou a diretriz elaborada, sem necessidade de ajustes posteriores.

Após o cálculo do IVC dos instrumentos de avaliação, procedeu-se à avaliação das sugestões dos painelistas. Foram, portanto, reunidas as sugestões e acatadas quando pertinentes (Figura 3). A consulta externa com as coordenadoras de enfermagem e médicas de todas as UTIs da instituição sede resultou em consenso e concordância a respeito dos pontos, tendo o IVC alcançado índice maior que 80% em todos os quesitos.

**Figura 3 -:** Alterações realizadas nos protocolos SEDE-E e SEDE-T após sugestões dos painelistas. Londrina, PR, Brasil, 2023

## Discussão

O ineditismo deste estudo consiste na elaboração, com base em evidências científicas, de dois protocolos clínicos de segurança para o manejo da sede: um para pacientes pós-extubados e outro para traqueostomizados, em uma abordagem inovadora. Permitem avaliar, por meio de critérios de segurança, a possibilidade de enfermeiros aplicarem, com menos de 24 horas de extubação e mesmo em traqueostomizados, a estratégia de alívio da sede de forma segura, abrindo espaço para o tratamento precoce da sede em pacientes que sofrem intenso distresse pela restrição hídrica imposta a essa fase terapêutica^(27)^.

Por não ser o método mais utilizado para a elaboração de protocolos clínicos, houve dificuldade para encontrar um instrumento de validação de conteúdo tanto para os fluxogramas como para os manuais operacionais. Desse modo, fez-se a avaliação por meio de ferramenta baseada nos domínios do *Appraisal of Guidelines for Research & Evaluation*, AGREE II, que avalia o rigor metodológico e a transparência de diretrizes. Ademais, os painelistas tiveram auxílio da ferramenta GRADEpro para formular o julgamento da recomendação^(31-32)^.

Os critérios de segurança elencados vão ao encontro de outros protocolos que têm como objetivo triar disfagia, como, por exemplo, o *Gugging Swallowing Screen Intensive Care Unit* (GuSS-ICU)^(34)^. Mesmo não sendo o foco desta pesquisa, evitar a broncoaspiração é um objetivo em comum. No fluxograma elaborado, inicia-se com os pré-requisitos, com o critério cabeceira elevada de 30° a 45°. Após a rodada Delphi, dois painelistas sugeriram ser critério de exclusão, porque há pacientes que apresentam impossibilidade de se elevar a cabeceira, por isso mais sujeitos ao risco de aspiração pulmonar.

O nível de consciência é, majoritariamente, o primeiro critério a ser avaliado. Outros estudos que objetivaram o alívio da sede^(1,20-21,35)^ ou avaliação de disfagia^(34)^ em pacientes críticos consideraram a avaliação deste parâmetro por meio de perguntas cognitivas simples^(1,20,35)^, Escala de Richmond de Agitação-Sedação (RASS)^(21,34)^ ou Escala de Coma de Glasgow (ECG)^(21)^. Estar acordado e em alerta, comunicando-se, mas não necessariamente orientado, não impede que o paciente possa receber estratégias de alívio da sede. Neste item, sugeriu-se a utilização da ECG, porém o CG, acrescido do suporte das chefias médicas e de enfermagem da instituição, considerou a ampliação do parâmetro da RASS, o que contemplaria, com suficiente segurança, o critério nível de consciência.

Como sugestão dos painelistas, incluiu-se oximetria de pulso em um critério mais amplo – insuficiência respiratória – para conferir maior segurança na avaliação. Também foram incluídos neste critério: dispneia, uso da musculatura acessória (tiragem intercostal, retração de fúrcula e abdominal), saturação de oxigênio <92% e presença de estridor laríngeo. Observa-se frequência respiratória, oximetria, presença de estridor como critérios relevantes em outros protocolos que avaliam a disfagia, porém não com esta nomenclatura específica^(34)^.

Ausência de náuseas e vômitos foi considerado critério essencial visando à prevenção de eventos adversos após a intervenção de alívio da sede. A broncoaspiração é a complicação mais temida referenciada por estudos que avaliam a reintrodução de líquidos e sólidos^(1,20-21,34)^, também fazendo parte de um protocolo de segurança que visa o manejo da sede em pacientes cirúrgicos^(36)^. Este critério obteve concordância total na avaliação do GP.

A tosse é essencialmente um mecanismo de proteção de via aérea^(35)^. Por meio deste recurso, garante-se que, se houver secreções ou corpos estranhos na extensão das vias aéreas superiores, serão expelidos. Em se tratando do manejo da sede, a tosse eficaz permitirá que a estratégia utilizada para o alívio da sede não obstrua as vias aéreas nem leve à broncoaspiração. Limpar a garganta intencional e vigorosamente por movimento característico é considerado suficiente para proteção de via aérea. Identifica-se este critério em pesquisas que visam ao manejo da sede e reintrodução alimentar^(1,21,34-35)^. Houve concordância de todos os juízes neste item.

Semelhantemente à tosse, a deglutição é um mecanismo de proteção de via aérea de extrema importância, sobretudo para o objetivo do presente estudo. Uma RS com metanálise mostrou que 41% dos pacientes pós-extubados apresentam disfagia, ocasionando aspiração pulmonar, sem diferenciação de intubação curta (<48 horas) ou prolongada (>48 horas)^(17)^. A triagem de disfagia com finalidade de fazer reintrodução alimentar é considerada essencial como medida protetiva^(34)^. O manejo da sede difere de introdução alimentar, ou seja, não retira o paciente do jejum, já que se tem como objetivo aliviar, de forma segura, o desconforto da sede por meio de substâncias frias e mentoladas com baixo volume.

Os painelistas apresentaram sugestões importantes em relação à avaliação do critério deglutição. Por conseguinte, após reunião com a fonoaudióloga, definiu-se que a deglutição seria avaliada pela elevação do osso hioide em conjunto com a solicitação de o paciente cerrar os lábios. Durante o processo da deglutição, o osso hioide, que fica acima da cartilagem laríngea, apresenta movimento de elevação superior e anterior^(36)^. Desse modo é possível avaliar, com o segundo e o terceiro dedos colocados acima deste osso, a elevação laríngea e, consequentemente, a deglutição^(37)^.

O último critério, no caso do SEDE-E, é alteração de voz. Apesar de ele não ter sido elencado por meio dos artigos da RS realizada, protocolos que visam avaliar o retorno da alimentação oral consideram a alteração de voz relevante^(34)^.

No SEDE-T não se incluiu alteração de voz devido à presença da cânula de traqueostomia, porém, introduziu-se o critério disfuncionalidade na cânula. Quando há disfuncionalidade no tubo em pacientes em uso de TQT, eles ficam mais propensos a eventos adversos ou pior prognóstico. Vazamento de cuff, pressão de cuff acima da recomendada, sangramentos, mal posicionamento e inconformidades com a ostomia são alguns exemplos^(37)^. Houve concordância de todos os juízes a respeito deste item.

As estratégias sugeridas para o manejo da sede foram: picolé de 20 mililitros, de gelo mentolado ou não, e spray mentolado gelado, ambos juntamente com o hidratante mentolado. A efetividade do picolé de gelo mentolado ou não tem sido demonstrada em diversos Ensaios Clínicos Randomizados (ECR)^(23,38-39)^, e o picolé vem sendo utilizado extensivamente por pacientes em Sala de Recuperação Anestésica^(39)^. Apenas um picolé de gelo mentolado reduz a intensidade da sede em 80,6%, e o desconforto em 75,9%^(39)^. Ressalta-se que o uso de gelo auxilia na recuperação da disfagia pela reativação da musculatura da deglutição^(40)^.

A temperatura fria e o mentol ativam receptores orofaríngeos, que enviam sinais diretamente à lâmina terminal, ocasionando a diminuição da vontade de beber água em aproximadamente dois minutos^(9,41-43)^. Ou seja, sem grandes volumes de líquido, sacia-se a sede e de forma mais rápida. Outra vantagem é que o derretimento do picolé é lento, o que amplia a segurança para o alívio da sede em comparação com a ingestão de água em volumes maiores.

O spray gelado e mentolado contempla os mesmos princípios de ativação dos termorreceptores orais e pode ser utilizado em pacientes que não conseguem tossir ou deglutir, têm alteração de voz ou estão com disfuncionalidade em cânula de TQT. Uma estratégia de alívio da sede baseada em spray é segura, pois é fácil de usar e distribui uniformemente pequenas gotículas de água (0,1 mililitro) na superfície mucosa da boca. Isso umedece a mucosa oral, controlando a quantidade de líquido introduzido e regulando a quantidade de líquido aplicado, o que, por sua vez, diminui possíveis reações adversas, proporcionando assim um elevado grau de segurança^(1)^.

Após as alterações e aprovação de duas doutoras especialistas em UTI, os fluxogramas SEDE-E e SEDE-T foram concluídos. Ambos os protocolos são um caminho para que pacientes pós-extubados e traqueostomizados tenham o sintoma tão angustiante e intenso da sede aliviado por um manejo simples.

Como limitação do estudo considera-se a realização de apenas uma rodada da Técnica Delphi, apesar de que a fragilidade foi superada mediante o consenso obtido com outro grupo de avaliadores, da medicina e enfermagem, com ampla experiência e expertise em terapia intensiva. O grupo de painelistas predominantemente de dois hospitais do Sul e Sudeste do Brasil é uma limitação, pois outras regiões do país podem ter perspectivas diferentes. Futuras avaliações dos protocolos em outros cenários contribuirão para testá-los em populações e realidades diferentes. Outra limitação foi a exiguidade de estudos que abordassem o manejo da sede na população de traqueostomizados, particularmente focando na avaliação de critérios de segurança, reduzindo a possibilidade de comparações. Sugere-se que a aplicação dos protocolos seja avaliada em cenários diversos, considerando-se variáveis intervenientes e de desfecho, assim como os benefícios percebidos pelo paciente e equipe de saúde.

O presente estudo contribui para a prática dos enfermeiros intensivistas, auxiliando na tomada de decisão e permitindo minorar a sede dos pacientes críticos com segurança, com base em evidências científicas. Além disso, desperta-se a necessidade de estudos nesta temática e da avaliação dos protocolos na prática clínica. Por fim, não só estimula os profissionais a voltarem seu olhar de forma intencional para a sede dos pacientes recém-extubados e traqueostomizados, mas também disponibiliza dois instrumentos que podem tornar possível aliviar com segurança a sede e o sofrimento de pacientes críticos.

## Conclusão

A elaboração de dois protocolos clínicos fundamentados em evidências, voltados para a segurança no manejo da sede em pacientes críticos pós-extubados e traqueostomizados, representa uma inovação e um avanço para o cuidado humanizado. Os protocolos propõem seis critérios de segurança cada: nível de consciência, sinais de insuficiência respiratória, náusea e/ou vômito, tosse eficaz, capacidade de deglutir e alteração de voz, e, no caso de traqueostomia, disfuncionalidade de cânula. Dispostos em fluxogramas de fácil compreensão, acompanhados por um manual operacional, os protocolos recém-desenvolvidos, além de terem validade de conteúdo, abrem as portas para a avaliação da segurança para o alívio da sede em pacientes críticos, contribuindo para a redução do distresse relacionado a esse sintoma.

# Translation: Elaboración de protocolos de seguridad para el manejo de la sed en pacientes post-extubados y traqueostomizados*

**DOI:** 10.1590/1518-8345.7376.4496

**Authors:** Isabela Bossi Faleiros, Meiriane Pizani Scobare de Oliveira, Aline Franco da Rocha, Lígia Fahl Fonseca

**Affiliations:** 1 Universidade Estadual de Londrina, Londrina, PR, Brasil

**Keywords:** Sed, Extubación Traqueal, Traqueostomía, Protocolos Clínicos, Guía de Práctica Clínica, Unidades de Cuidados Intensivos

## Abstract

elaborar, con base en evidencias, dos protocolos distintos con criterios de seguridad para pacientes post-extubados y traqueostomizados con el fin de respaldar la administración de métodos de alivio de la sed.

estudio metodológico dividido en dos fases: definición del alcance y elaboración de los protocolos. Se identificaron criterios de seguridad que fueron sometidos a evaluación de confiabilidad de las evidencias mediante el sistema *Grading of Recommendations, Assessment, Development and Evaluation* (GRADEpro). Los criterios fueron incorporados en flujogramas con sus respectivos manuales operativos. Los panelistas formularon recomendaciones y se calculó el Índice de Validez de Contenido.

se desarrollaron dos protocolos y sus respectivos manuales para la evaluación de seguridad en la administración de métodos de alivio de la sed: uno para pacientes post-extubados y otro para traqueostomizados. Criterios de seguridad enumerados: nivel de consciencia, signos de insuficiencia respiratoria, náusea y/o vómito, capacidad de deglución, tos/protección de las vías aéreas, alteración de la voz y funcionamiento adecuado de la cánula de traqueostomía. Se obtuvo recomendación de los panelistas con un Índice de Validez de Contenido del 87%.

los protocolos clínicos, con seis criterios de seguridad cada uno, basados en evidencias para el manejo de la sed en pacientes post-extubados y traqueostomizados, son innovadores, permiten la evaluación de seguridad para el manejo de la sed y tienen validez de contenido.

## Destacados

**(1)** La sed es intensa en pacientes post-extubados y traqueostomizados, pero no es tratada. **(2)** No existen protocolos con criterios de seguridad para manejar la sed en esta población. **(3)** Se elaboraron protocolos basados en evidencias con seis criterios de seguridad. **(4)** Los protocolos innovadores ayudan en la toma de decisiones del enfermero. **(5)** Permiten, mediante seis criterios de seguridad, la evaluación y manejo de la sed.

## Introducción

Diversas poblaciones de pacientes en restricción hídrica sufren de sed no tratada. Los pacientes internados en la Unidad de Cuidados Intensivos (UCI), en particular, presentan una alta prevalencia de intensidad y malestar por la sed, que varía de 69,8%^(1)^ a 76,1%^(2)^. Los principales factores que desencadenan este malestar incluyen: uso de opioides y diuréticos, medicamentos inhalatorios, niveles séricos de sodio y glucosa, capacidad de ingesta oral, ayuno, cirugías, osmolaridad y Ventilación Mecánica (VM)^(3-4)^.

La sed en el paciente crítico es prevalente y su manejo es desafiante; sin embargo, paradójicamente, con frecuencia es descuidada^(5-7)^. El equipo relaciona la sed principalmente con factores osmóticos y volumétricos (sed homeostática), enfocando su tratamiento únicamente en la rehidratación intravenosa. No obstante, la sed no homeostática se desencadena por estímulos de sequedad provenientes de la orofaringe, entre otros^(8-9)^, lo cual es particularmente relevante para su manejo en pacientes post-extubados y traqueostomizados.

La sed ocupa un papel principal en la percepción de estrés y sufrimiento de los pacientes en UCI. El uso de dispositivos como el tubo orotraqueal (TOT) y la necesidad de VM forman parte del día a día, lo cual provoca que la cavidad oral permanezca abierta y expuesta durante períodos prolongados. Debido a esto, los pacientes describen su experiencia de sed como una sensación abrumadora y constante, que se ha manifestado como prevalente^(10)^ e intensa^(5)^, siendo el principal y más aterrador factor estresante^(11)^, un síntoma angustiante^(12)^ y el segundo mayor malestar en la UCI^(13)^. Cuando el paciente está consciente y bajo VM, significa que es dolorosamente consciente del malestar que causa la sed^(14)^.

A medida que el cuidado intensivo se vuelve resolutivo, el paciente mejora, lo cual culminará en su extubación o en la sustitución del TOT por un dispositivo de larga permanencia, en este caso, la traqueostomía (TQT)^(15)^. Después de la extubación o durante el mantenimiento de una TQT en VM, es práctica clínica mantener al paciente en ayuno de sólidos y líquidos debido al riesgo de disfagia.

Este es un momento en el cual el paciente, tras haber estado intubado durante largos períodos, se queja insistentemente de sed, lo que hace urgente la necesidad de manejar este malestar. No existe consenso establecido en cuanto al momento ideal para la realización de pruebas que confirmen la presencia de disfagia, con prácticas que varían de 6 hasta 24 horas de ayuno. Sin embargo, estos procedimientos carecen de estandarización y no siguen directrices claras basadas en evidencias^(16)^.

La complicación más preocupante al considerar la introducción temprana de alimentos líquidos y sólidos es la disfagia post-extubación (DPE), que afecta al 41% de los adultos gravemente enfermos. Esta contribuye al aumento de las tasas de neumonía, prolongando la estancia en la UCI y generando mayores costos de atención en salud^(17)^.

Es necesario desarrollar protocolos que contrarresten la disfagia y hagan posible el alivio de la sed en pacientes críticos. Algunas estrategias que utilizan sustancias frías y mentoladas^(1,18-25)^ han demostrado efectividad en el alivio de la sed en pacientes críticos. No obstante, no se ha identificado en la literatura la existencia de protocolos que permitan a los enfermeros en las UCI evaluar la seguridad en la utilización de estrategias para el manejo de la sed, particularmente para los pacientes recién extubados y traqueostomizados.

La elaboración de un protocolo clínico basado en evidencias sólidas fue esencial para guiar el cuidado, posibilitando el manejo temprano de la sed en estas dos poblaciones. La Práctica Basada en Evidencias (PBE) representa un enfoque metodológico que promueve el uso de intervenciones eficaces y exitosas en la práctica clínica, al integrar las mejores evidencias científicas provenientes de investigaciones, experiencia clínica y de relevancia para el paciente, con el objetivo de fundamentar la toma de decisiones^(26)^.

Aunque existan estrategias efectivas para mitigar la sed, es fundamental, antes de emplear algún método de alivio, determinar los criterios de seguridad, especialmente en pacientes recién extubados o con traqueostomía. Ante las experiencias en entornos clínicos y las lagunas en la literatura, se hizo evidente la necesidad de elaborar un protocolo basado en evidencias científicas que permita el uso seguro y temprano de métodos para aliviar la sed. El objetivo de este estudio fue elaborar, con base en evidencias, dos protocolos distintos con criterios de seguridad para pacientes con TOT y TQT a fin de respaldar la administración de métodos de alivio de la sed.

## Método

### Delineamiento del estudio

Se trata de un estudio metodológico con enfoque cuantitativo. La elaboración de protocolos clínicos se basa en datos sólidos y bien fundamentados, lo que resulta en información confiable y validada^(27)^.

Como referencia metodológica, se utilizó el manual “Directrices Metodológicas: elaboración de directrices clínicas” del Ministerio de Salud, que consta de dos fases: alcance y elaboración. La primera fase consiste en la definición del tema y del grupo de trabajo y en la construcción de la estrategia de búsqueda de evidencia. La segunda fase, a su vez, comprende la realización de la estrategia de búsqueda, extracción, análisis de las evidencias, opinión de expertos para el análisis de las evidencias y la elaboración de recomendaciones mediante el Método Delphi y, finalmente, la elaboración de la directriz basada en evidencias^(27)^.

### Participantes

Para desarrollar los protocolos, se delimitaron diferentes grupos encargados de organizar el proceso, definir el alcance, coordinar el equipo de trabajo, redactar el documento, resumir las evidencias y elaborar recomendaciones. Estos grupos incluyeron individuos con experiencia en el área y especialistas que determinaron las evidencias. De esta forma, para llevar a cabo la investigación, se constituyeron los siguientes grupos: Comité Gestor, Grupo Elaborador y Grupo de Panelistas^(27)^.

El Comité Gestor (CG) estuvo compuesto por el investigador principal, su orientador y co-orientador, responsables de la operacionalización del proceso. El Grupo Elaborador (GE) se conformó con la unión del CG y el investigador secundario, quien fue responsable de aplicar la estrategia de búsqueda y elaborar la directriz. El Grupo de Panelistas (GP) estuvo conformado por enfermeros, médicos y fisioterapeutas intensivistas, un fonoaudiólogo y técnicos de enfermería, encargados de participar en la definición de recomendaciones, ayudar en la interpretación de los resultados, formular sugerencias y revisar el contenido de la directriz.

Para la selección del GP, se consideraron profesionales de la salud con más de tres años de experiencia en cuidados intensivos. Se invitó a 17 profesionales, por medio de carta física y medio electrónico, incluidos siete enfermeros, dos fisioterapeutas, un fonoaudiólogo, cinco médicos y dos técnicos de enfermería. Participaron en la investigación 16 profesionales, ya que un técnico de enfermería declinó la invitación.

### Definición del tema y estrategia de búsqueda

El tema definido para la directriz fue seguridad para el manejo de la sed en pacientes post-extubados y traqueostomizados en UCI. Se estableció el tema a partir de la demanda observada por los autores y de solicitudes al Grupo de Estudio e Investigación de la Sed (GPS), con sede en la *Universidade Estadual de Londrina* (UEL). Una vez propuesto el tema, la directriz fue elaborada en dos etapas, siguiendo el referente metodológico propuesto. La primera fue enumerar y analizar las evidencias mediante una revisión sistemática (RS) con metaanálisis titulada “Prevención de broncoaspiración en el manejo de la sed en pacientes críticos: revisión sistemática y metaanálisis”.

La RS siguió la metodología *Preferred Reporting Items for Systematic Reviews and Meta-Analyses* (PRISMA)^(28)^ y recibió el número de registro CRD42023429760 en la plataforma PROSPERO, donde los datos de registro indican rigor metodológico en el protocolo de búsqueda. El artículo de revisión, sin embargo, generó resultados extensos y, por lo tanto, no pudo incluirse en este constructo. La búsqueda se realizó el 14 de junio de 2023. Los artículos seleccionados presentaron bajo riesgo de sesgo según las herramientas Rob 2^(29)^ y Robins I^(30)^, y alta certeza de evidencia por medio del *software* GRADEpro^(31)^. Bajo este rigor metodológico, fue posible enumerar seis criterios de seguridad para la elaboración de una directriz basada en evidencias para el manejo de la sed en pacientes post-extubados y traqueostomizados, los cuales fueron: nivel de consciencia, protección de las vías aéreas mediante la capacidad de deglutir y toser, oximetría de pulso y ausencia de náuseas/vómito.

### Elaboración del protocolo

Aunque no se hayan catalogado en los artículos de la RS, se añadieron a los criterios descritos: alteración de la voz y disfuncionalidad de la traqueostomía. El primero, por estar contemplado en protocolos de cribado de disfagia y reintroducción alimentaria; el segundo, debido al alto riesgo de adversidades por posibles anomalías en la TQT.

Seleccionados los criterios, se procedió a la segunda etapa del estudio, con la elaboración de dos flujogramas y sus respectivos manuales operativos. Los protocolos se denominaron SEDE-E (extubados) y SEDE-T (traqueostomizados) para diferenciar el flujograma en relación con la población objetivo. Se optó por elaborar dos protocolos debido a la especificidad de cada situación clínica: los pacientes recién extubados no presentan dificultad de deglución por la permanencia de la vía aérea definitiva, mientras que los pacientes traqueostomizados presentan menor riesgo de broncoaspiración debido a la protección del *cuff* inflado.

### Validación y recomendación de la evidencia

Para la validación de contenido, se emplearon instrumentos separados para los dos flujogramas. En el primer instrumento, Validación de Contenido del Flujograma, se evaluaron cinco dimensiones: impresiones generales sobre el flujograma, diseño, contenido del flujograma, aplicabilidad y pertinencia del contenido del flujograma con el manual operativo. Cada dimensión estaba compuesta por ítems que explicaban lo que debía evaluarse en ella, y los panelistas realizaron juicios sobre cada uno de los ítems. Esta herramienta fue adaptada de los dominios del *Appraisal of Guidelines for Research & Evaluation*, AGREE II^(32)^. Las respuestas comprendían una escala Likert de cinco puntos: totalmente en desacuerdo, en desacuerdo, ni de acuerdo ni en desacuerdo, de acuerdo y totalmente de acuerdo, seguidas de consideraciones y sugerencias libres de los panelistas. Los ítems evaluados en las dimensiones fueron utilizados para el cálculo del Índice de Validez de Contenido (IVC), considerándose favorables las puntuaciones de “de acuerdo” y “totalmente de acuerdo”.

El segundo instrumento, Validación de Contenido del Manual Operativo, consistió en juzgar cada ítem del manual en cuanto a su objetividad (permite una respuesta puntual), claridad (explicado de manera clara, simple e inequívoca) y pertinencia (evalúa la seguridad para el manejo de la sed), seguido de sugerencias para reformulación. Cada ítem de la herramienta fue utilizado para el cálculo del IVC, y se consideró favorable la respuesta “sí” para cada juicio.

El tercer instrumento fue el Juicio de la Recomendación de la Intervención. Esta herramienta se compuso de varios ítems cuyo objetivo era ayudar al panelista a realizar su recomendación. Al final de la evaluación, la recomendación podía ser juzgada según lo estipula el GRADEpro: recomendación fuerte en contra de la intervención, recomendación condicional en contra de la intervención, recomendación condicional para la intervención o la comparación, recomendación condicional para la intervención, recomendación fuerte para la intervención^(31)^. Se consideraron favorables la recomendación condicional para la intervención y la recomendación fuerte para la intervención.

El cuarto y último fue la caracterización socio-ocupacional de los panelistas en cuanto a: edad, profesión, tiempo de actuación, grado de escolaridad, tipo y complejidad de institución en la que trabaja y campo de actuación. La información sirvió para trazar el perfil de los participantes. Concluida la elaboración de los flujogramas y manuales operativos, los respectivos archivos fueron enviados por medio electrónico a los panelistas – tras aceptar su participación –, con las debidas orientaciones de los autores respecto a la lectura y el desarrollo de la evaluación. El autor permaneció disponible para aclarar dudas que no implicaran un impacto en el rigor metodológico. Para alcanzar consenso entre ellos, se utilizó el Método Delphi^(27)^, en una ronda, en el período de julio de 2023. Las devoluciones de los panelistas fueron tabuladas en una hoja de cálculo de Excel^®^ (Versión 2308). El cálculo del IVC fue determinado considerando la representatividad de cada ítem y debía alcanzar un índice de concordancia del 80%(33), condición para que el CG aceptara las sugerencias pertinentes.

Después de las sugerencias de los panelistas, se consultó a una fonoaudióloga especialista en disfagia, con quince años de experiencia en UCI. Sus sugerencias, junto con las de los panelistas, contribuyeron a la formulación de un proceso para evaluar, de manera directa y sistemática, la capacidad de deglutir. Además, después de las modificaciones, se realizó una consulta técnica externa con la coordinadora de enfermería y la jefatura médica de la división de terapia intensiva de la institución del estudio, quienes validaron todas las modificaciones sintetizadas por el CG, tal como establece el Manual de Directrices^(27)^.

La metodología propuesta por el manual incluye la consulta con panelistas con el fin de obtener consenso del grupo en relación con la interpretación de las evidencias disponibles, aunque el manual indique que, en la jerarquía de las evidencias para la toma de decisiones en salud, la opinión de expertos se considera de nivel bajo debido a su mayor susceptibilidad a sesgos. Además, recomienda el sistema GRADE por ofrecer una metodología amplia y transparente para la toma de decisiones estructurada, siendo esta la metodología preferida por el Ministerio de Salud^(27)^.

De esta forma, el objetivo de la consulta a los panelistas fue la recomendación de las evidencias ya recopiladas, con fuerte indicio de aprobación. Dado que las evidencias pasaron por el sistema GRADEpro^(31)^, no demostraron sesgo mediante las herramientas Robins I^(30)^ y Rob 2^(29)^, todas las sugerencias fueron aceptadas, y el protocolo fue recomendado con un índice del 87% en el IVC. El CG juzgó, entonces, que no era necesaria una nueva ronda Delphi de evaluación con el GP.

## Aspectos éticos

El estudio respetó los aspectos éticos relacionados con la investigación en seres humanos, de acuerdo con lo establecido en la Resolución CNS 466/12. El proyecto de investigación fue presentado al Comité de Ética en Investigación de la *Universidade Estadual de Londrina* y obtuvo la aprobación ética bajo el número CAAE: 65456622.2.0000.5231.

## Resultados

Se desarrollaron dos flujogramas basados en evidencias científicas para el manejo de la sed, respetando las particularidades de cada grupo de pacientes. El primer flujograma fue dirigido a pacientes post-extubados, SEDE-E (Figura 1); el otro, a traqueostomizados, SEDE-T (Figura 2). Los pacientes post-extubados ya no presentan la dificultad de deglución relacionada con el TOT y su *cuff*; mientras que los traqueostomizados, por su parte, permanecen con la cánula traqueal y su *cuff* inflado.

Los flujogramas fueron elaborados junto con sus respectivos manuales operativos. Los manuales ayudan en la aplicación del protocolo y explican cada criterio de seguridad, orientando la toma de decisiones.

**Figura 1 -:** SEDE-E: Flujo de manejo de la sed en el paciente post-extubado. Londrina, PR, Brasil, 2023

**Figura 2 -:** SEDE-T: Flujo de manejo de la sed en el paciente traqueostomizado. Londrina, PR, Brasil, 2023

Participaron 16 panelistas: siete enfermeros, dos fisioterapeutas, un fonoaudiólogo, cinco médicos y un técnico de enfermería. La caracterización sociodemográfica de los panelistas se describe en la Tabla 1.

**Tabla 1 -:** Distribución de las características sociodemográficas y profesionales de los participantes del estudio (n = 16). Londrina, PR, Brasil, 2023

**Variables**	**n**	**%**
**Formación profesional**		
Enfermería	07	43,7
Medicina	05	31,2
Fisioterapia	02	12,5
Fonoaudiología	01	06,3
Técnico en Enfermería	01	06,3
**Formación educacional**		
Curso Técnico	01	06,3
Especialización	04	25,0
Maestría	05	31,2
Doctorado	06	37,5
**Tamaño de la institución de trabajo**		
Especial (más de 500 camas)	03	18,7
Gran tamaño (151-500 camas)	12	75,0
Mediano tamaño (51–150 camas)	01	06,3
**Edad**		
Hasta 31 años	01	06,3
Entre 31 a 40 años	08	50,0
Entre 41 a 50 años	03	18,7
Más que 50 años	04	25,0
**Tiempo de actuación profesional**		
Entre 5 y 10 años	03	18,7
Entre 11 y 15 años	03	18,7
Entre 16 y 20 años	01	06,3
Entre 21 y 25 años	02	12,5
Entre 26 y 30 años	01	06,3
Más que 31 años	04	25,0
No respondieron	02	12,5

La Validación de Contenido del Flujograma para el SEDE-E mostró que el ítem sobre el orden de los criterios alcanzó un IVC de 0,75 de concordancia; por lo tanto, se modificó el orden de los criterios a una secuencia más lógica. La dimensión sobre el contenido del manual operativo, tanto en SEDE-E (0,68) como en SEDE-T (0,62), no alcanzó el 80% de concordancia, por lo que se sugirieron modificaciones para mejorar la evaluación de la deglución (Tabla 2).

**Tabla 2 -:** Índice de validez de contenido del instrumento de validación de contenido del flujograma y manual operativo. Londrina, PR, Brasil, 2023

**Dimensiones**	IVC* SEDE-E	IVC* SEDE-T
IVC* Validación de Contenido del Flujograma		
**Impresiones Generales**		
Su uso es fácil	1,00	1,00
Es autoexplicativo	1,00	1,00
Es didáctico	1,00	1,00
**Diseño del Flujograma**		
La composición visual es atractiva y está bien organizada	0,87	0,87
El orden de los criterios de seguridad es adecuado	0,75	0,87
El flujograma es de fácil lectura	0,93	1,00
Los colores utilizados en el diseño del flujograma son pertinentes	0,93	0,93
**Contenido del Flujograma**		
Los criterios de seguridad indicados en los rombos del flujograma están claros	0,81	0,81
Los criterios de seguridad se presentan de manera organizada en una secuencia correcta	0,87	0,81
**Aplicabilidad del Flujograma**		
Las recomendaciones del flujograma tienen aplicabilidad práctica	0,87	0,81
**Contenido del Manual Operativo**		
El contenido es coherente con el flujograma	0,93	0,87
La información es adecuada	0,75	0,81
La información es suficiente para el uso del flujograma	0,68	0,62
IVC* Validación de Contenido del Manual Operativo		
**Prerrequisitos**		
Objetivo	1,00	0,87
Claro	0,93	0,62
Pertinente	0,87	0,81
**Examen preliminar**		
Objetivo	1,00	1,00
Claro	0,87	0,93
Pertinente	1,00	1,00
**Nivel de consciencia**		
Objetivo	1,00	1,00
Claro	1,00	1,00
Pertinente	0,81	0,81
**Oximetría de pulso**		
Objetivo	1,00	1,00
Claro	0,93	0,93
Pertinente	0,81	0,81
**Náusea y/o vómito**		
Objetivo	1,00	1,00
Claro	0,93	0,93
Pertinente	1,00	1,00
**Deglución eficaz**		
Objetivo	0,87	0,87
Claro	0,75	0,81
Pertinente	0,87	0,87
**Alteración de voz**		
Objetivo	0,93	-
Claro	0,87	-
Pertinente	1,00	-
**Disfuncionalidad con la cánula de traqueostomía**		
Objetivo	-	0,93
Claro	-	0,87
Pertinente	-	0,93
**Tos y/o limpiar garganta eficaz**		
Objetivo	0,93	0,93
Claro	0,93	0,87
Pertinente	1,00	0,93
**Estrategia de manejo da sede**		
Objetivo	1,00	0,93
Claro	0,93	0,87
Pertinente	0,93	0,93

*IVC = Índice de Validación de Contenido

El instrumento de Validación de Contenido del Manual Operativo obtuvo una evaluación satisfactoria, con solo un criterio por debajo del 80% en el IVC, en el SEDE-E, referente a la claridad sobre cómo evaluar la deglución; se sugirió mejorar la forma de evaluar la deglución. En cuanto a la evaluación del SEDE-T en este instrumento, el ítem de claridad en los prerrequisitos obtuvo un valor de 0,62, debido a un error de diagramación en la explicación de este manual; se aceptó la sugerencia y se corrigió el flujograma (Tabla 2).

El resultado del último instrumento – Juicio de la Recomendación de la Intervención – alcanzó un IVC del 87% a favor de la intervención con los protocolos SEDE-E y SEDE-T. En vista de ello, el GP recomendó la directriz elaborada, sin necesidad de ajustes adicionales.

Después de calcular el IVC de los instrumentos de evaluación, se procedió a evaluar las sugerencias de los panelistas. Se recopilaron las sugerencias y se aceptaron cuando fueron pertinentes (Figura 3). La consulta externa con las coordinadoras de enfermería y las médicas de todas las UCI de la institución sede resultó en consenso y conformidad respecto a los puntos, logrando un IVC superior al 80% en todos los criterios.

**Figura 3 -:** Modificaciones realizadas en los protocolos SEDE-E y SEDE-T tras sugerencias de los panelistas. Londrina, PR, Brasil, 2023

## Discusión

La originalidad de este estudio radica en la elaboración, basada en evidencias científicas, de dos protocolos clínicos de seguridad para el manejo de la sed: uno para pacientes post-extubados y otro para traqueostomizados, en un enfoque innovador. Permiten evaluar, mediante criterios de seguridad, la posibilidad de que enfermeros apliquen estrategias de alivio de la sed de forma segura en menos de 24 horas tras la extubación e incluso en pacientes traqueostomizados, abriendo espacio para el tratamiento temprano de la sed en pacientes que experimentan un gran malestar debido a la restricción hídrica impuesta en esta fase terapéutica^(27)^.

Dado que este no es el método más utilizado para la elaboración de protocolos clínicos, resultó difícil encontrar un instrumento de validación de contenido tanto para los flujogramas como para los manuales operativos. Por ello, se utilizó una herramienta basada en los dominios del *Appraisal of Guidelines for Research & Evaluation*, AGREE II, que evalúa el rigor metodológico y la transparencia de las directrices. Además, los panelistas contaron con el apoyo de la herramienta GRADEpro para formular el juicio de recomendación^(31-32)^.

Los criterios de seguridad enumerados son consistentes con otros protocolos que buscan evaluar la disfagia, como el *Gugging Swallowing Screen Intensive Care Unit* (GuSS-ICU)^(34)^. Aunque no era el enfoque de esta investigación, la prevención de la broncoaspiración es un objetivo compartido. En el flujograma elaborado, se inicia con los prerrequisitos, con el criterio de cabecera elevada a 30°-45°. Tras la ronda Delphi, dos panelistas sugirieron que este fuera un criterio de exclusión, ya que algunos pacientes no pueden elevar la cabecera y, por lo tanto, tienen mayor riesgo de aspiración pulmonar.

El nivel de conciencia es, en su mayoría, el primer criterio a evaluar. Otros estudios que abordan el alivio de la sed^(1,20-21,35)^ o la evaluación de la disfagia^(34)^ en pacientes críticos han considerado este parámetro mediante preguntas cognitivas simples^(1,20,35)^, la Escala de Agitación-Sedación de Richmond (RASS)^(21,34)^ o la Escala de Coma de Glasgow (ECG)^(21)^. Estar despierto y alerta, aunque no necesariamente orientado, no impide que el paciente reciba estrategias de alivio de la sed. En este ítem, se sugirió el uso de la ECG, pero el CG, junto con el apoyo de las jefaturas médicas y de enfermería de la institución, consideró suficiente ampliar el parámetro de la RASS, lo cual garantiza la seguridad en el criterio de nivel de conciencia.

Como sugirieron los panelistas, se incluyó la oximetría de pulso dentro de un criterio más amplio de insuficiencia respiratoria para una mayor seguridad en la evaluación. También se añadieron a este criterio: disnea, uso de musculatura accesoria (tiraje intercostal, retracción de la fúrcula y abdominal), saturación de oxígeno <92% y presencia de estridor laríngeo. La frecuencia respiratoria, la oximetría y la presencia de estridor son criterios relevantes en otros protocolos de evaluación de disfagia, aunque no con esta terminología específica^(34)^.

La ausencia de náuseas y vómitos se consideró un criterio esencial para prevenir eventos adversos tras la intervención de alivio de la sed. La broncoaspiración es la complicación más temida en estudios que evalúan la reintroducción de líquidos y sólidos^(1,20-21,34)^, y también forma parte de un protocolo de seguridad para el manejo de la sed en pacientes quirúrgicos^(36)^. Este criterio obtuvo una total concordancia en la evaluación del GP.

La tos es esencialmente un mecanismo de protección de las vías aéreas^(35)^. Por medio de este reflejo, se asegura que, si hay secreciones o cuerpos extraños en las vías aéreas superiores, estos sean expulsados. En el contexto del manejo de la sed, una tos eficaz permite que la estrategia utilizada para aliviar la sed no obstruya las vías aéreas ni provoque broncoaspiración. La acción de aclarar la garganta de manera intencionada y vigorosa es suficiente para la protección de las vías aéreas. Este criterio se identifica en estudios sobre manejo de la sed y reintroducción alimentaria^(1,21,34-35)^ y contó con el consenso de todos los jueces en este ítem.

Al igual que la tos, la deglución es un mecanismo esencial de protección de las vías aéreas, sobre todo para el objetivo de este estudio. Una RS con metaanálisis mostró que el 41% de los pacientes post-extubados presentan disfagia, lo que provoca aspiración pulmonar, independientemente de si la intubación fue corta (<48 horas) o prolongada (>48 horas)^(17)^. La evaluación de la disfagia con el fin de reintroducir la alimentación es considerada esencial como medida protectora^(34)^. El manejo de la sed difiere de la reintroducción alimentaria, ya que no interrumpe el ayuno, sino que busca aliviar de manera segura el malestar de la sed mediante el uso de sustancias frías y mentoladas en bajo volumen.

Los panelistas presentaron importantes sugerencias sobre el criterio de evaluación de la deglución. Tras una reunión con la fonoaudióloga, se definió que la deglución sería evaluada observando la elevación del hueso hioides mientras se pide al paciente que cierre los labios. Durante la deglución, el hueso hioides, situado sobre la laringe, se eleva en dirección superior y anterior^(36)^. De esta manera, es posible evaluar la elevación laríngea y, en consecuencia, la deglución, colocando el segundo y tercer dedos sobre este hueso^(37)^.

El último criterio en el caso del SEDE-E es la alteración de la voz. Aunque no fue un ítem encontrado en los artículos de la RS realizada, los protocolos que evalúan el regreso a la alimentación oral consideran relevante la alteración de la voz^(34)^.

En el SEDE-T no se incluyó la alteración de voz debido a la presencia de la cánula de traqueostomía; en su lugar, se introdujo el criterio de disfuncionalidad de la cánula. Cuando hay disfunción del tubo en pacientes con TQT, estos tienen mayor riesgo de eventos adversos o mal pronóstico. Ejemplos de esto incluyen fugas de *cuff*, presión de *cuff* por encima de lo recomendado, sangrados, mala posición y problemas en la ostomía^(37)^. Este ítem también contó con la concordancia de todos los jueces.

Las estrategias sugeridas para el manejo de la sed fueron paleta de hielo de 20 mililitros mentolada o no, y spray frío mentolado, ambos en combinación con hidratante mentolado. La efectividad de la paleta de hielo mentolada o no ha sido demostrada en diversos Ensayos Clínicos Aleatorizados (ECA)^(23,38-39)^, y ha sido utilizada extensamente por pacientes en la Sala de Recuperación Anestésica^(39)^. Una sola paleta de hielo mentolada reduce la intensidad de la sed en un 80,6% y el malestar en un 75,9%^(39)^. Cabe destacar que el uso de hielo ayuda en la recuperación de la disfagia al activar los músculos de la deglución^(40)^.

La temperatura fría y el mentol activan los receptores orofaríngeos, enviando señales directamente al sistema nervioso central, lo cual reduce la necesidad de beber agua en aproximadamente dos minutos^(9,41-43)^. Esto permite saciar la sed sin grandes volúmenes de líquido y de forma más rápida. Otra ventaja es que el derretimiento de la paleta es lento, lo que aumenta la seguridad para el alivio de la sed en comparación con la ingesta de agua en mayores volúmenes.

El spray frío mentolado comparte los mismos principios de activación de los termorreceptores orales y puede ser utilizado en pacientes que no pueden toser o deglutir, tienen alteración de voz o presentan disfuncionalidad en la cánula de TQT. Una estrategia de alivio de la sed basada en spray es segura, ya que es fácil de usar y distribuir pequeñas gotas de agua (0,1 mililitro) de manera uniforme en la mucosa bucal, lo cual humedece la mucosa oral y controla la cantidad de líquido aplicado, reduciendo posibles reacciones adversas y proporcionando un alto grado de seguridad^(1)^.

Tras las modificaciones y la aprobación de dos doctoras especialistas en UCI, los flujogramas SEDE-E y SEDE-T fueron concluidos. Ambos protocolos son un recurso para que pacientes post-extubados y traqueostomizados puedan aliviar el intenso y angustiante síntoma de la sed mediante un manejo simple.

Como limitación del estudio, se considera la realización de solo una ronda de la Técnica Delphi, aunque esta debilidad fue compensada mediante el consenso alcanzado con otro grupo de evaluadores de medicina y enfermería con amplia experiencia en terapia intensiva. El grupo de panelistas, mayoritariamente de dos hospitales del sur y sureste de Brasil, representa una limitación, ya que otras regiones del país pueden tener diferentes perspectivas. Futuras evaluaciones de los protocolos en otros contextos contribuirán a su validación en poblaciones y realidades diferentes. Otra limitación fue la escasez de estudios sobre el manejo de la sed en la población de traqueostomizados, especialmente en relación con la evaluación de criterios de seguridad, lo cual limitó las posibilidades de comparación. Se sugiere evaluar la aplicación de los protocolos en diversos escenarios, considerando variables intervinientes y resultados, así como los beneficios percibidos por los pacientes y el equipo de salud.

Este estudio contribuye a la práctica de los enfermeros intensivistas, apoyando la toma de decisiones y permitiendo reducir de manera segura la sed de los pacientes críticos, basada en evidencias científicas. Además, destaca la necesidad de realizar estudios en esta temática y de evaluar los protocolos en la práctica clínica. Finalmente, no solo incentiva a los profesionales a prestar una atención intencional a la sed de los pacientes recién extubados y traqueostomizados, sino que también pone a disposición dos instrumentos que pueden permitir aliviar de manera segura la sed y el sufrimiento de los pacientes críticos.

## Conclusión

La elaboración de dos protocolos clínicos basados en evidencias, orientados a la seguridad en el manejo de la sed en pacientes críticos post-extubados y traqueostomizados, representa una innovación y un avance en el cuidado humanizado. Estos protocolos proponen seis criterios de seguridad cada uno: nivel de consciencia, signos de insuficiencia respiratoria, náusea y/o vómito, tos eficaz, capacidad de deglutir y alteración de voz, y, en el caso de traqueostomía, disfuncionalidad de la cánula. Dispuestos en flujogramas de fácil comprensión, acompañados de un manual operativo, los protocolos recién desarrollados, además de contar con validez de contenido, abren posibilidades para evaluar la seguridad en el alivio de la sed en pacientes críticos, contribuyendo a reducir el malestar asociado a este síntoma.
